# Prevalence of cardiac involvement in sarcoidosis detected by CMR: Initial results in 90 patients

**DOI:** 10.1186/1532-429X-15-S1-P151

**Published:** 2013-01-30

**Authors:** Daniel Thomas, Andreas Goebel, Jonas Doerner, Darius Dabir, Dirk Skowasch, Stefan Pabst, Hans H Schild

**Affiliations:** 1 Department of Radiology, University of Bonn, Bonn, Germany; 2 Department of Pulmonology, University of Bonn, Bonn, Germany

## Background

Autopsy studies report a cardiac involvement in sarcoidosis as high as 20-60%. Clinically a much lower detection rate of approximately 5% has been reported. Since cardiac events are the second most frequent cause of death, a high rate of undetected cases of cardiac involvement can be presumed, potentially leading to a fatal event as the fist manifestation. Previous studies have shown the superiority of CMR to detect cardiac involvement in sarcoidosis compared to other imaging modalities or clinical scores. We aimed at investigating the prevalence of previously unknown cardiac involvement in sarcoidosis in a caucasian population.

## Methods

The local institutional review board approved this prospective study. Patients were recruited through a nationwide patient support group and the local pulmonology department.

Only patients with histologically proven sarcoidosis, but without previously known cardiac involvement following routine clinical assessment were included. The CMR protocol included SSFP cine images covering the entire left ventricle, early relative contrast enhancement, T2 STIR imaging in the short axis orientation as well as delayed enhancement imaging covering the standard cardiac axes. Two experienced readers, blinded to all clinical data, analyzed the CMR studies.

**Figure 1:**
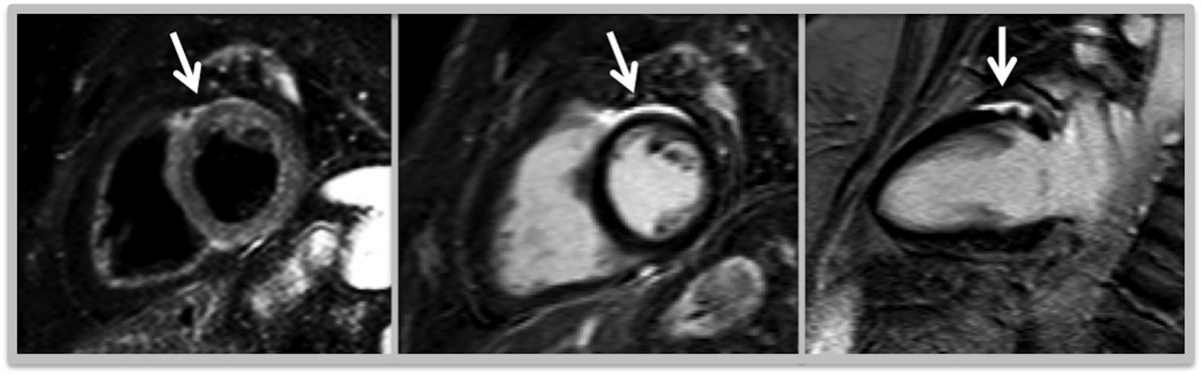
From left to right (arrows): Short axis T2 Stir showing edema. Short and vertical long axis showing necrosis.

## Results

During the first recruitment period from August 2011 through February 2012 a total of 90 patients were included into the study (58 females) with a mean age of 52 years (median 52).

The mean duration since diagnosis of sarcoidosis was 115 months (median 80 months). In this population extranodal and extrapulmonary manifestation were present in the skin (22.2%), in the liver (8.8%), in the eye (16.6%) and in the central nervous system (7.7%). Global cardiac function parameters were as follows: LVEDV 112ml, IVSD 10mm, LVEF 68%.

CMR revealed previously unknown cardiac involvement in 7/90 Patients (7.8%) (figure demonstrating typical enhancement pattern and regional edema in one patient). The diagnosis was based on the presence of typical delayed enhancement (n=5) or early gadolinium enhancement plus wall motion abnormality or pericardial effusion. Patients with cardiac involvement did not demonstrate a reduction of global cardiac function compared to the patients without cardiac involvement: LVEDV 111ml, IVSD 10mm, LVEF 69%.

## Conclusions

In this preliminary study in 90 patients CMR detected seven cases of cardiac involvement in sarcoidosis (7.8%), missed by standard clinical testing. This finding suggests a potential role for CMR in the routine workup of sarcoidosis patients, but warrants further investigation to determine the impact of CMR findings on patients` prognosis.

## Funding

n.a.

